# An Overview of the Use of the SimSphere Soil Vegetation Atmosphere Transfer (SVAT) Model for the Study of Land-Atmosphere Interactions

**DOI:** 10.3390/s90604286

**Published:** 2009-06-03

**Authors:** George Petropoulos, Toby N. Carlson, Martin J. Wooster

**Affiliations:** 1 University of Bristol, Department of Earth Sciences, Wills Memorial Building, Queens Road, BS8 1RJ, Bristol, UK; 2 Pennsylvania State University Department of Meteorology, University Park, PA 16802, USA; E-Mail: tnc@meteo.psu.edu (T.N.C.); 3 King's College London, Department of Geography, London, WC2R 2LS, UK; E-Mail: martin.wooster@kcl.ac.uk (M.J.W.)

**Keywords:** land surface simulation process models, Soil Vegetation Atmosphere Transfer (SVAT) models, SimSphere model overview

## Abstract

Soil Vegetation Atmosphere Transfer (SVAT) models consist of deterministic mathematical representations of the physical processes involved between the land surface and the atmosphere and of their interactions, at time-steps acceptable for the study of land surface processes. The present article provides a comprehensive and systematic review of one such SVAT model suitable for use in mesoscale or boundary layer studies, originally developed by [1]. This model, which has evolved significantly both architecturally and functionally since its foundation, has been widely applied in over thirty interdisciplinary science investigations, and it is currently used as a learning resource for students in a number of educational institutes globally. The present review is also regarded as very timely, since a variation of a method using this specific SVAT model along with satellite observations is currently being considered in a scheme being developed for the operational retrieval of soil surface moisture by the US National Polar-orbiting Operational Environmental Satellite System (NPOESS), in a series of satellites that are due to be launched from 2016 onwards.

## 1. Introduction

Surface-Vegetation-Atmospheric Transfer (SVAT) models are mathematical representations of vertical ‘views’ of the physical mechanisms controlling energy and mass transfers in the soil/vegetation/atmosphere continuum, providing deterministic estimates of the time course of soil and vegetation state variables at time-steps compatible with the dynamics of atmospheric processes [2]. The development of SVAT models has been described by [3] as essentially the result of the convergence of the following needs; i) the requirement for better information and understanding on land surface processes, ii) the need for information useful to determine how plants and plant communities respond to different and changing environmental conditions, and iii) the general goal of every hydrological model which is the functional requirement to provide boundary conditions or help assess surface hydrological balance.

Compared to other approaches currently employed to study surface-atmospheric transfers, such as remote sensing, use of SVAT models has several advantages but also some limitations. One of their strongest advantages includes their fine time-step (usually less than one hour) that is in satisfactory agreement with the timescale of the physical processes being simulated. In addition, SVAT models are able to provide detailed description usually of a large number of soil and vegetation canopy processes, and not only of a limited number of final variables such as latent (LE) and sensible (H) heat fluxes or net primary production (e.g. [4]). However, as reported by [5], the degree of realism of a SVAT model generally depends on the models structural complexity (i.e. the detail in the model physics), the representativeness and configuration of the different components (such as the number of soil layers), the quality of the input data used for parameterisation, and also on the site-specific conditions such as local climate, biophysical and geophysical characteristics and their ability to be represented by the parameterisations contained within the model. Use of SVAT models is also generally constrained by their requirement for a large array of input parameters, sometimes resulting in difficult initialisations [6]. Furthermore, since these models are generally one-dimensional vertical representations, they have little or no representation of horizontal transport and are limited in their capacity to produce spatially distributed outputs. This is a key reason why many studies have focused more recently on the combined use of SVAT models with remote sensing data, as it allows merging of the horizontal coverage, repetitive nature and high spatial detail of satellite remote sensing with the vertical coverage and temporal continuity of the SVAT model.

By linking such 1D SVAT models to the spatialised information provided by airborne or satellite earth observation (EO) data, a potentially more powerful synergistic avenue has been developed to take advantage of the benefits of both the modelling and EO-based approaches [10]. Overviews of some of the numerical and data assimilation approaches linking remote sensing to hydrological models, including SVAT models, can be found in [7,8] and recently in [9], whereas [2] provides a comprehensive discussion on the variety of methods used for incorporating remote sensing data specifically into SVAT models. A convenient categorisation of these can be found in [2]: (i) “forcing” methods, where the model is fed directly with quantities derived from remotely sensing – such as surface temperature or soil moisture content (e.g. [11]); (ii) “recalibration” methods, whereby certain of the SVAT model input parameters are adjusted based on minimisation of the difference between their simulated values and those derived from remote sensing (e.g. [4,12]); (iii) the “special recalibration” methods, also called as the “triangle” methods, originally proposed by [13] and later modified by [14]. The “triangle” approach is based on a contextual interpretation of a EO-derived scatter plot of the surface temperature (T_s_) and vegetation index (VI) related data, linked to simulations from the “SimSphere” SVAT model (the name of its most recent implementation) and designed to produce spatially distributed estimates of the LE and H fluxes as well as of surface soil moisture availability (M_o_). A recent overview of the workings of this “triangle” approach can be found in [15]. However, an overview of the use of this SVAT model, which here for clarity we refer to as the “SimSphere” model by the name of its most recent implementation, both as a standalone application and via its coupling to remotely sensing data in the “triangle” method and elsewhere, is at present somewhat lacking from the literature. This despite the extensive use of this specific SVAT model by the scientific community in a large number of interdisciplinary science studies [16-19], and its exploitation also as an educational resource in a number of Universities worldwide (e.g. Pennsylvania State University and University of Purdue in the USA). A variant of the “triangle” method which may embed the use of the SVAT model, is also being considered for use in an operational application of the “triangle” method, as part of the surface soil moisture retrieval scheme developed for the US National Polar-orbiting Operational Environmental Satellite System (NPOESS), commencing in 2016 [20].

In the context of the above developments, the objective of the current article is to review the utility and use of the SimSphere SVAT model, both in terms of an overview of the models architecture and development (and without extensive reference to mathematics and/or technical jargon), summarize and classify its main application areas (each of which have evolved considerably since the its initial implementation by [1]), and derive a series of conclusions from the model use to date. The article complements as well the study of [15], which focused solely on the principles and limitations of the “triangle” method alone. In this context, Section 2 provides a synoptic description of the SVAT model architecture (without extensive reference to mathematics and/or technical jargon), Section 3 makes available a comprehensive review of the models application areas, whilst Section 4 provides a series of conclusions derived from the model use to date.

## 2. Overview of the SVAT Model Architecture

This section provides a descriptive account of the SVAT model architecture, based on the most recent implementation by [14], termed “SimSphere”. The aim here is to provide a non-mathematical description, free of technical jargon, which allows the reader to understand the basic principles of the model architecture. Further systematic description of this architecture and the model initialisation procedure can be found in [21] as well as in the online e-learning site where the model is currently hosted (http://www.e-education.psu.edu/simsphere/). An extensive mathematical account of the basis of the model has been provided previously by [1,22], whilst the model's bare soil component is described by [22], its vegetation component by [16,23], and its representation of plant hydraulics by [24-26]. The most recent version of the SVAT model is freely available from the Department of Meteorology of Pennsylvania State University, USA (https://courseware.e-education.psu.edu/simsphere/ or http://www.agry.purdue.edu/climate/dev//simsphere.asp).

**Figure 1.**
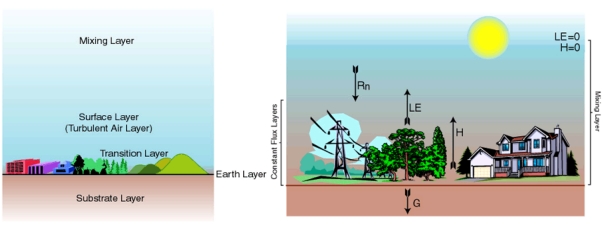
In the left figure are shown the different layers of the SimSphere SVAT model in the vertical domain, whereas the figure on the right provides a schematic representation of the surface energy balance components computation in SimSphere. R_n_ is the net radiation, LE, H and G are the latent, sensible and ground heat fluxes respectively. Figure adapted from the SimSphere user's manual (available at https://courseware.e-education.psu.edu/simsphere/workbook).

The SimSphere SVAT model is essentially a one-dimensional boundary layer model with a plant component (Figure 1). In the horizontal domain, the scale of the model implicitly represents a horizontal area of the Earth's surface of undefined size that can be considered to be composed of a mixture of bare soil and vegetation, in proportions (F_r_) and (1-F_r_) varying from 0 to 1.0, where F_r_ being the fractional vegetation cover per unit area. Thus, it is conceivable that the horizontal scale of the model is defined by the degree to which the model's initial conditions (i.e. input parameters) are representative of the horizontal area to be simulated. Simsphere applies to a point or, at least, a limited region as long as the atmospheric, surface slope and incident radiation are uniformly distributed over the domain. SimSphere has been developed to simulate the various physical processes that take place as a function of time in a column that extends from the root zone below the soil surface up to a level higher than the surface vegetation canopy. The model performs simulations over a 24-hour cycle, starting from a set of initial conditions given in the early morning (at 05:30 hours local time) and simulates the continuous evolving interaction between soil, plant and atmospheric layers. SimSphere requires a number of parameters for its initialization (53 in total), divided into seven groups: time and location, vegetation, surface, hydrological, meteorological, soil and atmospheric. From a set of initial parameters representative of the site conditions on which the model is implemented, a number of prognostic variables (32 in total) are calculated from the SVAT model including the surface energy fluxes (H, LE and ground heat flux) at the soil surface, and in, around and above the vegetation canopy, the flux of carbon dioxide between the atmosphere and the plants, the grid-cell scale integrated surface temperature (T_s_) of the vegetation and soil mixture.

**Figure 2.**
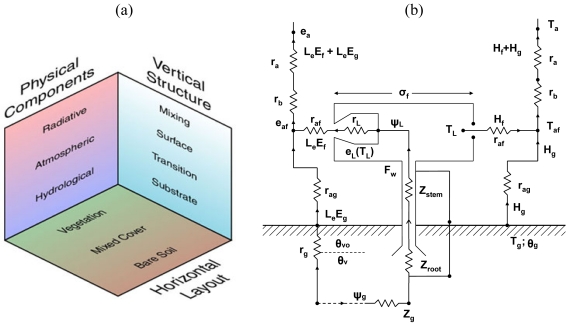
The figure on the left (a) depicts the different facets of the SVAT model architecture (figure adopted from SimSphere user's manual (available at https://courseware.e-education.psu.edu/simsphere/workbook). The figure on the right (b) is a synthesis of the figures provided by [23,24] and provides a representation of the vertical structure of the plant-canopy model in the form of an electrical analogy illustrating how the exchange of the LE and H fluxes between plant, atmosphere and surface is represented in the model. Definition of the individual model parameters described on Figure 2b is provided in Table 1.

The different facets of the SVAT model's overall structure, namely the physical, the vertical and the horizontal, are illustrated in Figure 2. The overall model structure is expressed in terms of resistances as an Ohm's Law analogue (Figure 2b), with parameters referred to in Table 1. The different components of each of the model facets are described briefly below. The depths of the different model layers are variable with time. The top of the mixing layer is allowed to rise during the day in response to H fluxes from the surface, and is identified by the presence of a temperature inversion that caps the air in convective contact with the surface layer. The surface layer, or turbulent air layer, extends from the top of the bare soil transition layer (or from the top of the vegetation layer) to a height of 50 m. The transition layer, a somewhat mathematical ‘fiction’, applies only to the vertical transfer of heat and moisture over the bare soil component, and governs the flux of heat and momentum in a hypothetical layer from the roughness height for heat to the roughness height for momentum. In the case of vegetation, the transition layer is replaced by a vegetation layer. The substrate layer refers to the depth of the soil over which heat and water are conducted. In the model, soil water content is specified by the user for two layers, a surface layer and a root zone layer, by assigning a fractional volume of field capacity.

As depicted in Figure 2a, the model planetary boundary layer (PBL) (i.e. the layer of atmosphere whose behavior is directly influenced by its contact with the planetary surface) consists of a vegetated surface fraction (F_r_) and a bare soil fraction. Each of these two regimes is treated separately, but the fluxes are blended at the base of the surface layer, nominally set at 50 m height. The PBL receives most of its heat and virtually all of its water vapour from the surface through the vertical transfer of heat, momentum, water vapour and carbon dioxide. This is accomplished primarily by turbulent eddies that are driven by surface heating, and by mechanical turbulence produced by wind between the surface and the atmosphere. The underlying constraint in the model is that the energy fluxes at the Earth's surface and within the plant canopy must balance appropriately. Initial forcing of the model begins with the calculation of solar radiation, determined from a one-dimensional solar radiation model. Details of the solar and long wave radiative transfer models are provided by [27]. Briefly, the total down-welling irradiance absorbed in the substrate layer is calculated from the solar geometry, atmospheric transmission coefficients and the surface albedo for the particular date, time and latitude/longitude location being simulated. Calculation of the radiation fluxes by default assumes cloud-free conditions, though an adjustment for a set percentage cloud cover can be made in the initialisation, if required.

As regards the vegetation parameterisation of the SimSphere SVAT model (Figure 2b), when the vegetation component is activated the model accounts for a layer of vegetation between the atmospheric surface layer and the ground. Vegetation density is expressed in terms of Leaf Area Index (LAI) (that being the one sided green leaf area per unit ground area) and F_r_. When combining the separate bare soil and vegetation representations to account for conditions of partial vegetative cover, at the level of the canopy, the bare soil and vegetation regimes operate separately, but are allowed to interact through exchanges of momentum, heat and water vapour with the common surface and mixing layers above the canopy and the common substrate below (Figure 2b).

The shortwave incoming radiation and downward long-wave radiation are calculated in an identical way for the bare soil and vegetation regimes, and the radiation partitioning is computed as a function of the foliage density. In a similar way, the LE and H fluxes and the upward flux of long-wave radiation above the plant canopy and the substrate heat flux (G) are taken in the SVAT model as averages of the bare soil and vegetation components, weighted according to the vegetation fraction. Temperature and specific humidity at the top of the surface layer (T_a_; q_a_), and the temperature and water content in the soil, are identical for both bare soil and vegetation fractions. Computation of canopy T_s_ is conducted from the weighted average of the bare soil and vegetation components of upward long-wave radiation fluxes.

A fundamental part of the vegetation parameterisation constitutes the modelling of plant stomatal resistance. This is because essentially this parameter in the model expresses the resistance of vegetation to transpire and also because it plays a key role in the regulation of energy partitioning between LE and H fluxes. The SVAT model used in the present study allows the choice between two stomatal resistance parameterisations either the [28] or the [25] formulation. The first parameterisation has the advantage that it is able to capture the gross aspects of stomatal behaviour as affected by soil water and sunlight, but its dominant disadvantage is that it ignores plant hydraulics, which accounts for significant shifts in transpiration rate over the diurnal period. In [25], stomatal resistance is expressed as a product of a dimensionless function that describes the effect of incident solar flux, leaf water potential and vapor pressure deficit on the stomatal resistance. Stomatal resistance in this type of parameterization is also a function of leaf-atmosphere vapor pressure difference. The latter is expressed as the difference between the mesophyl and epidermal leaf water potentials and the stomatal effect is modeled as being proportional to the vapor pressure difference. In this last type of stomatal resistance parameterisation, the product of these functions is scaled to the units of resistance, by multiplying by a factor termed the minimum stomatal resistance, which is set as a constant for each simulation. An important aspect of the [25] stomatal resistance formulation is the imposition of a threshold epidermal water potential, below which stomatal resistance increases rapidly with decreasing leaf water potential. When the threshold leaf water potential is reached, the transpiration tends to remain nearly constant until the leaf water potential again crosses the threshold toward higher values. [25] have termed this “flattening” of the transpiration curve the “transpiration plateau”.

In terms of scaling the simulated parameters from leaf to canopy, the calculations by the SVAT model are made initially for a single ‘big leaf’, in a similar way to other SVAT model approaches (e.g. the “DAISY” model of [29]). Simulated fluxes are expressed in units of Watts per m^2^ of leaf area, in order that they can be related to the surface energy balance. Conversion from flux per unit leaf area to flux per unit surface area is made by scaling the fluxes by the leaf area index divided by a “shelter factor” as defined in [30]. The shelter factor accounts for the fact that not all leaves transpire at the sunlit amount due to the fact that available solar radiation decreases with height beneath the top of the canopy. A detailed description of the scaling factor concept employed in the model can be found in [16,24].

## 3. Overview of SVAT Model Use

The present section reviews the use and development of the [1] SimSphere SVAT model to date, it having evolved considerably since its initial implementation. It is generally difficult to classify these methods since the SVAT model has been exploited in a large number of interdisciplinary studies. However, for convenience and efficiency, we propose a categorisation of the SVAT models use into the following three main groups, based on the focus of the study in question:

1. Evaluation of the land surface parameters simulated by the SVAT model, including sensitivity analysis studies
2. Use of the SVAT model as a tool to explore hypothetical scenarios and perform other analysis studies
3. Coupling of the SVAT model with remote sensing data (for example in the so-called “triangle approach” of [14,31] to which reference was made earlier).

### 3.1. Studies based on the evaluation of the land surface parameters simulated by the SVAT model, including sensitivity analysis studies

A large number of studies relating to the use of the SimSphere SVAT model have been concerned with the evaluation of the coherence of the model structure and of its ability to provide realistic simulations of key land surface parameters. Such studies have included either direct comparison between model simulations and actual ground observations, or comparisons to other SVAT model simulations, and also some sensitivity analysis experiments. [1] first compared selected model outputs derived from simulations of an early version of the SVAT model at both urban and rural sites located in USA and Australia under cloud-free conditions. In their study, excellent agreement was obtained in the diurnal patterns of the simulated LE and H fluxes and T_s_ between the simulated values and the observations. However, the magnitudes of the observed fluxes appeared to be overestimated by the model, though no absolute quantitative measure of this overestimation was made available by the authors. One limitation was that the results were based in an early version of the SVAT model, which did not include the vegetation component. A sensitivity analysis was conducted in [1], and underlined the importance of M_o_ and thermal inertia as the two most sensitive model input parameters in terms of accurately simulating T_s_ and the soil heat flux profile. In addition, the authors reported the atmospheric turbidity, wind speed, ground albedo and surface roughness as other important, albeit less critical, model input parameters for the simulation of the H flux and T_s_. However, the sensitivity analysis study performed by the authors had been based on a rather simple variation of selected model inputs from a baseline value, and observation of the resultant changes in the considered outputs.

**Figure 3.**
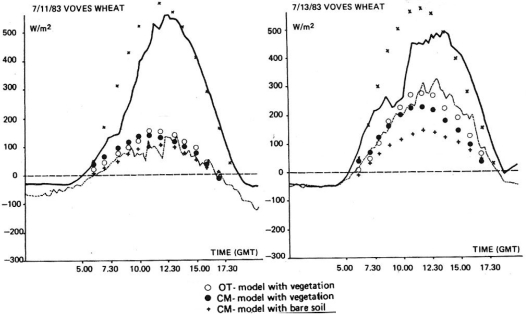
Measured R_n_ flux (shown with solid lines) and surface H flux (shown with dashed lines) and H fluxes simulated by the [16] (OT) and [31] (CM) models with and without the vegetation component (● is the H flux predicted by the OT model with vegetation parameterisation, ○ is the H flux predicted by CM model with vegetation and × is the H flux predicted by the CM model without vegetation parameterisation included). Figure adapted from [16].

[16] evaluated the ability of the boundary layer models of [32] and [16] in simulating H flux and soil moisture using each boundary layer model with and without the vegetation components. In their study, verification of both models was conducted using observational data from a wheat crop site located in France for selected cloud-free days as well with concurrent T_s_ measures derived from Advanced Very High Resolution (AVHRR) satellite data. Overall, although a good agreement with ground measurements of the energy fluxes was found, higher divergences were observed when the vegetation model was activated in the two models. The latter was attributed by the authors to the tight control exerted by the stomatal resistance term on the calculated fluxes in the vegetation model, and to the different treatment of thermal inertia in the two models. In the same study [16] also performed a relatively simple sensitivity analysis, concluding that errors in the model initialisation and specifically in the sounding temperature and wind speed, dewpoint sounding and geostrophic winds can dramatically affect the simulated surface fluxes and soil moisture (Figure 3). Their results also showed that the thermal inertia significantly impacts the simulation of both the substrate heat fluxes and of maximum T_s_, a finding that was in agreement with [1].

In [33] comparable simulations between three urban boundary layer models were performed, including the SVAT model of [22], for the same initial conditions obtained from a climatological station in Los Angeles, USA. Differences in the model outputs which were attributed to the different model architectures and parameterisation schemes. Specifically the [22] model was reported to show too great a proportion of R_n_ being used to drive LE flux, and too little by H flux, which resulted in average daytime Bowen ratios that were too small for urban environments. However, [33] reported that between all models considered in their study, the model of [22] was the one that produced the most reasonable energy budget, as a result of its more realistic boundary layer structure. Nonetheless, it should be underlined that authors reached this last conclusion based on comparisons of the simulated fluxes to representative values reported in the literature, and not to actual observations.

Similarly to [33], [34] evaluated the ability of three energy balance models - including that of [1] - in simulating key components of the surface energy budget in an urban environment (a city suburb of Canada). Notably, results from their work indicated that none of the examined models was able to provide consistent predictions of the turbulent fluxes of LE and H, which the authors attributed to the inability of the different models to handle the role of water availability. Also, from all the compared models in the [34] study, the SVAT of [1] was able to provide the best R_n_ flux prediction, albeit with a consistent overestimation of the ground heat flux (G). Sensitivity analysis experiments conducted by the same authors identified the most critical model inputs for the simulation of the energy fluxes, M_o_, thermal inertia and heat capacity, followed by the meteorological input parameters (wind speed, vapour pressure and climatic mean temperature).

[24] included in the SVAT model of [22] the use of a stomatal resistance model that also had the ability to incorporate an analytical solution for leaf water potential. The novelty of their proposed stomatal resistance model was that it allowed for epidermal control of leaf water potential by specifying a gradient of leaf water potential between the surface and the interior of the leaf, where that gradient was dependent of the vapour pressure deficit. Subsequently, authors demonstrated the consistency of the SVAT model performance for a corn crop using data from one summer cloud-free day from the HAPEX field experiment in France [35]. The same authors also reported results from a series of sensitivity analysis tests to the SVAT model which suggested a clear dependence of leaf water potential, stomatal resistance, transpiration and T_s_ on M_o_, root-stem resistance, critical leaf water potential and “β” (beta). The “β” parameter relates to the difference between epidermal and mesophyl leaf water potentials, and makes stomatal resistance sensitive to vapour pressure deficit (i.e. the atmospheric dryness, in effect). However, again, their sensitivity analysis experiments were based on simple variations of selected model inputs to the outputs examined, rather than a more sophisticated approach that would allow deriving quantitative measures of the sensitivities of the model inputs and of their interactions with respect to the simulation of the output examined each time. [25] further modified the SVAT model of [24], including the plant capacitance model which had a similar structure to those of [36-38]. In the same study, authors also examined the effects of stored water (the capacitance) in the transpiration flux, T_s_, stomatal resistance and leaf water potential. For this purpose they used the same atmospheric conditions as in the studies of [24,13] and in-situ observations taken for a corn crop on a summertime day from the French Lubbon site. An important finding related to the demonstration of the significant role of capacitance on the surface energy budget only during periods of plant water stress, modifying leaf water stress and the transpiration plateau, affecting also the canopy T_s_.

[39] used ground observations from an agricultural (corn) site in the USA to investigate the ability of the SVAT model of [25] to simulate conditions of canopy transient water stress (expressed by the presence of a plateau in the evapotranspiration diurnal variation and the higher canopy T_s_). Threshold leaf water potential model input was found to have a key importance in the simulation of the canopy transient water stress, which was reproduced effectively by the SVAT model. Another important finding related to the appearance of the plateau, which using the SVAT model, the authors demonstrated may even occur when the soil is relatively moist provided that the atmospheric and solar demands are high. Lastly, a cursory sensitivity analysis was used by the authors to demonstrate that vegetation fractional cover and cloud cover are two of the model input parameters that dramatically affect the simulation of the transpiration plateau.

[26] employed ground observations from an agricultural soybean crop site in France to examine the ability of the SVAT models of [25,16] to simulate the midday depression in photosynthesis and the transpiration plateau. The latter was simulated as a consequence of imposing a threshold water potential in stomatal and photosynthesis regulations. Their comparisons indicated in general a good agreement for both photosynthesis and sap flow comparisons for the two models. In the same work, authors also examined the sensitivity of different model inputs on the diurnal evolution of transpiration and photosynthesis, illustrating a variety of behavior that can occur in different plants. A significant finding of their sensitivity analysis study was related to the use of the “β” parameter (previously referred to as the link between vapour pressure deficit and stomatal resistance) in the model of [25] as one of the controlling model input parameters in reproducing the control of leaf water potential for different types of plants. Although, this finding was in agreement with the results reported by [24], their sensitivity testing was also based on a relatively simple method. However, a more appropriate quantitative sensitivity analysis approach to the SVAT model, as for instance is done with global sensitivity analysis (GSA) methods, has not been performed by the model users. GSA methods allow a quantitative assessment of the sensitivity all model input parameters (not just one or two parameters) to the considered model output, including their interactions of the inputs rather than studying only the effects of individual/specific model parameters.

### 3.2. Studies based on the use of the SVAT model as a tool to explore hypothetical scenarios and perform other analysis studies

[40] used the SVAT model of [22] to investigate the correlations between airborne-derived T_s_, M_o_ and model-simulated and microwave soil water measurements for a flat agricultural region of short vegetation mostly subtle, grass) in France. Authors using the SVAT model showed that M_o_ varied almost linearly with measured microwave soil water content and that correlations between measured soil water contents and those derived from infrared temperature improved as the variance in the measurements increased. [40] concluded that correlations between soil moisture and infrared temperature become very low if the standard error of infrared temperature measurements over the field is less than ± 2 °C.

[41] used a more recent version of the SVAT model by [31], - essentially the same version of the model as that of [25] but with a more user-friendly interface interface and coded in C++ rather than fortran programming language - to investigate the potential effect of the doubling of ambient CO_2_ concentration on plant transpiration on corn and soybean crops grown in typical well-watered mid-latitude summertime conditions. Their findings, which were in contrast with laboratory studies such as [42], suggested that doubling of atmospheric CO_2_ would be expected to generate a negligible change in transpiration for these crops despite an increase in stomatal resistance associated with the CO_2_ increase. These differences were attributed by the authors to gaps in the understanding of the feedback processes, and thus to the exclusion of these in the SVAT model representation.

[43] employed the same version of SVAT model in another theoretical study, examining the feedback processes on evapotranspiration on two crops (soybean C3 and corn C4), grown under three different atmospheric CO_2_ concentrations over three full growing seasons in a region in the USA. Their simulations, which we note were consistent with the conclusions of others [41,44], showed that for these agricultural crops the surface layer feedback is large and stomatal control of transpiration significantly reduced. Simulations with the SVAT model suggested soil evaporation to be particularly important in decreasing the effects of CO_2_ increase on evapotranspiration, whereas a moderate feedback was found to result from the interactions of stomatal conductance and the simulated canopy microclimate. Seasonal dynamics (i.e. observed increase in leaf area at elevated CO_2_ concentrations) was also reported to affect the feedback processes, but to a far lesser degree than stomatal conductance.

[18] used the PSUBAMS version of the SVAT model of [31] to investigate the response of soybean (C3) and corn (C4) plants to changes in stomatal resistance and transpiration under different environmental conditions, for both present day and doubled atmospheric CO_2_ concentrations. For this purpose they used the in-situ data already available from the works of [41,43], as well as the most recent version of the SVAT model [14] and the GENESIS general circulation model [45]. Results from both models also suggested that the increase of stomatal resistance which was observed in conditions of doubled atmospheric CO_2_ is likely to result in only minor decreases in transpiration and insignificant changes in surface energy balance. The same authors also underscored the importance of the mixing layer inclusion, which was present in the model of [14], in order the model to be able to represent the additional negative feedbacks that further reduced the sensitivity of transpiration to an increase in stomatal resistance.

**Figure 4.**
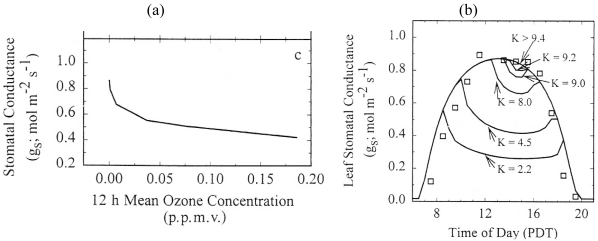
(a). Modelled relationships between 12h seasonal mean ozone (O_3_) concentration during plant development and midday stomatal conductance to water vapour of exposed transpiring leaves. (b). Representative diurnal course of stomatal conductance (g_s_) measured in a well-irrigated field of cotton (□) and modelled with non-limiting hydraulic conductance (K > 9.4 mmol m^-2^ MPA^-1^ s^-1^) corresponding to low ambient exposure to O_3_ during plant development (outer, bell-shaped curve). Increasing simulated exposures to O_3_ (successively lower K) progressively reduces modelled maximum g_s_ through increasingly severe midday stomatal closure (successively lower lines). Figures adapted from [46].

[46] employed the SVAT model and ground observations from a cotton plant in a site in USA, to test the hypothesis that ozone-induced reduction of hydraulic phase (K) is sufficient to mediate dose-dependent reductions in leaf stomatal conductance (g_s_), reproducing the linear relationship between vapour phase conductance to water flux (G_s_) and hydraulic phase (K) conductance to water flux. As described in more detail in [46] ozone interaction was included in the SVAT simulations as a function of stomatal resistance, leaf mesophyll and epidermal water potential and input of local environmental parameters after a modification of the stomatal resistance model originally developed by [25], allowing simulating ozone concentration from the bulk soil up to the atmospheric mixed layer of the model. The SVAT model reproduced the experimentally determined, statistically significant linear correlation between midday G_s_ and K (Figure 4a), maintaining the homeostasis of simulated midday leaf water potential (Ψ_m_). [46] also reported a close agreement between the simulated and observed canopy-scale fluxes of water and O_3_ under low ambient O_3_ concentrations (Figure 4b), underscoring also the importance of the critical water potential to the canopy-scale fluxes of water vapor and O_3_.

From a different perspective, [47] utilised the SVAT model which included the updates described by [16] to illustrate the sensitivity of the “B” and “n” empirically adjusted constant of the [48] and later modified [49] 24 hour integrated evapotranspiration (LE_24_) equation, expressed as:

(1) $$R_{n24}-LE_{24}=B{\left(T_{s}-T_{\alpha ir}\right)}^{n}$$

where R_n24_ is the 24-hours integrated net radiation, T_s_ is the radiometric surface temperature, T_air_ is the air temperature and “B” and “n” are empirically adjusted constants.

[47] in the same study also proposed a modified version of the [48,49] equation for deriving the daily evapotranspiration from remotely sensed surface temperature and minimal meteorological data. The authors used simulations from the SVAT model to determine the values for the equation coefficients, where model constants were obtained over a range of surface roughness and wind speeds, and where the equation had the following form:

(2) $$R_{n24}-LE_{24}=B^{\prime }{\left(\Delta \text{T}/\Delta t\right)}^{n^{\prime }}$$

where ΔT/Δt is an average rate of temperature rise during the morning (in °C h^-1^) and B′ and n′ are constants. According to the authors the advantage of their proposed method was that it did not require ground-based T_air_ measurements. However, it should be noted that authors did not demonstrate use of the new formulation with actual remote sensing observations.

[50] continued the work of [47] by proposing a simplified equation for the retrieval of the daily evapotranspiration, according to which the “B” and “n” parameters were derived as a function of the normalised (i.e. scaled) Normalized Difference Vegetation Index (NDVI), the actual surface temperature and the T_air_ (the latter derived from the T_s_/VI remote sensing data scatterplot). [50] also employed the SVAT model and observations from selected AVHRR images and two aircraft images from NS001 sensor taken from three locations in USA and Europe to examine relationships between NDVI and the parameters of the [48] equation. Their results were in agreement with those of [47], indicating that the “B” and “n” parameters vary systematically with F_r_ and NDVI but also vary to a lesser degree with wind speed and surface roughness. An important advantage of their proposed methodology, was its dependence on a very small number of easily obtainable parameters derived primarily from remote sensing data (namely, T_s_, T_air_ and R_n_ expressed as an integrated value over a 24-hours period).

### 3.3. Studies based on the coupling of the SVAT model to remote sensing observations

In addition to the studies reviewed thus far, the SVAT model has also been quite widely used in combination with remotely sensed. [1] were the first to suggest a numerical/graphical SVAT model inversion method for the retrieval of M_o_ and thermal inertia from a set of satellite derived temperatures made at different times during the day together with an initial guess of the values of M_o_ and thermal inertia. Later [22], using as a basis the method of [1], proposed an approach allowing the derivation of spatially explicit maps of the LE and H fluxes, as well as M_o_ and thermal inertia (P). Briefly, their method was based on the combination of the SVAT model and satellite-derived surface temperatures taken at two different times of the day, ideally close to the times of temperature maximum and minimum. The details of their methodology are described in [22] and the authors demonstrated the applicability of their proposed methodology over two regions of the USA using the SVAT model and two Heat Capacity Mapping Mission (HCMM) night/day image pairs. Their analysis showed good agreement in the spatial patterns of the regional maps of the turbulent fluxes and M_o_ for both the urban and rural areas, albeit no quantitative comparisons were performed. [51] also worked on illustrating the use of the method of [22] in one urban and one rural area in the USA, using also T_s_ data from HCMM. They highlighted the potential use of the energy fluxes derived via the latter method for the estimation of plume dispersion.

**Figure 5.**
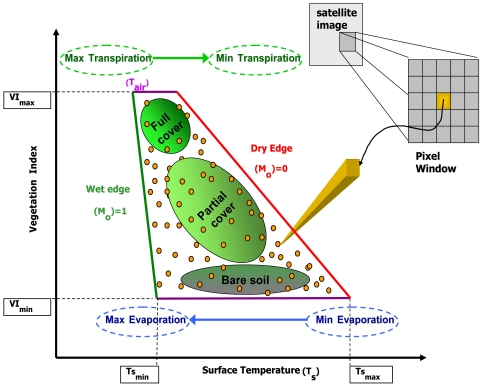
Summary of the main physical properties and interpretations of the satellite (or airborne) derived T_s_/VI feature space. Dots represent measurements at pixels observed by a VIS/TIR radiometer at various vegetation fractional covers (F_r_) and surface temperatures (T_s_). In this illustration, pixels classified as water or clouds are assumed to have been masked out. Figure is the product of a synthesis of figures presented by [57-59].

A significant amount of work has also been conducted coupling simulations from the reviewed here SVAT model to remote sensing data, based on a methodology that has its basis on the physical properties represented by a satellite-derived two-dimensional scatter plot of surface temperature (T_s_) versus vegetation index (VI). Provided that a full variation of both parameters exists in the scene, and that areas of standing water and clouds have been masked out, such a scatter plot has been demonstrated to often produce a characteristic “triangular”, or to some “trapezoidal”, shape - similar to that shown in Figure 5, which summarises the most important physical properties of the T_s_/VI scatterplot which are relevant to the estimation of the surface heat fluxes and M_o_.

The properties of this triangular T_s_/VI domain have been established by a large number of studies dating back to the early 1980's ([13,52,53]), and associations have been made between this pixel envelope and the estimation of various land surface parameters including heat fluxes and soil moisture content [54-56]. The emergence of the triangular (or trapezoid) shape in T_s_/VI feature space is the result of the low sensitivity of T_s_ to M_o_ variations over areas covered by vegetation, but its increased sensitivity (and thus larger spatial variation) over areas of bare soil. In the representation shown in Figure 5, each yellow circle within the T_s_/VI feature space represents the measurements from a single image pixel, assuming that cloud-contaminated pixels and those containing water have been masked out from the remotely sensed dataset. In Figure 5 the right-hand side border of the triangle (or trapezoid) (the so-called “dry or warm edge”) is defined by the locus of points of highest temperature but which contain differing amounts of bare soil and vegetation and which is assumed to represent conditions of limited surface soil moisture content. Likewise, the left hand border (the so-called “wet or cold edge”) corresponds to the set of cooler pixels that have varying amounts of vegetation and which represent those pixels at the limit of maximum soil water content. The remaining points within the triangular space correspond to pixels with varying vegetation index (i.e. F_r_), between those with bare soil and those with dense vegetation. The presence of a trapezoidal, rather than perfectly triangular, shape in the T_s_/VI feature space plot, results from a variation in soil thermal inertia with changing soil water content (which affects the soil heat storage and therefore the soil temperature). Various studies have also been concerned with the study of parameters affecting the shape of the T_s_/VI domain. [57,58] summarise as the main factors to be the F_r_, the thermal properties of scene components (temperature, moisture content), the synoptic state of the atmosphere (T_air_ and vapour pressure deficit), as well as the net radiation (R_n_), atmospheric forcing, M_o_ and the physiological activity of vegetation.

[50] and later [31] were one of the first to propose the use of a SVAT model of [31] in combination with satellite-derived estimates of T_s_ and VI for the retrieval of the surface fluxes and surface soil moisture availability from the T_s_/VI scatterplot domain. The so-called “triangle” approach, is principally based on a reduction of the satellite datasets to F_r_ and surface temperature (T_s_) parameters which are then coupled with simulations from the SVAT model parameterised for the test site conditions. The equations for the retrieval of M_o_ as well as the energy heat fluxes are subsequently derived for the time of the sensor overpass through a contextual interpretation of the triangular domain which emerges from the T_o_/F_r_ scatterplot. These equations are finally applied to the satellite datasets to invert for the instantaneous and daytime average fluxes (expressed as the ratio of LE or H to R_n_, which authors claimed to be less dependent on the time of day and more intrinsically related to M_o_ and F_r_) as well as the M_o_ on the sensor pixel scale. A more detailed description of the workings of the “triangle” method can be found in [14,60] whereas an overview of the method principles and of its limitations was made available recently by [15]. [32] demonstrated the application of the “triangle” method using the SVAT model of [17] and AVHRR data for a test region located in the United Kingdom. However, in their study, authors did not provide any quantitative results concerning neither the SVAT performance nor the “triangle” method derived outputs. Instead, their assessment was based on a visual comparison between of their computed spatially distributed maps derived from the “triangle” method versus the spatial variability patterns across the terrain of their study area. [14] performed a validation of the “triangle” method using also the SVAT model of [31]. Comparisons versus observations collected from the FIFE [61] and MONSOON 90 [62] field campaigns showed standard errors between derived and measured LE and H fluxes were reported to be between 25 and 55 Wm^-2^ (or about ±10% and ±30% respectively) of the magnitude of the fluxes, and for M_o_ about 16%. [63] for a region in the USA compared satellite derived M_o_ from selected AVHRR data using the “triangle” method and the SVAT model of [30]. These were compared versus profile ground measurements of M_o_, obtained from a soil hydrology model developed by [64]. Their results showed the satellite–derived surface moisture to be consistently underestimated with respect to those derived from the hydrological model. Agreement between the compared M_o_ parameters for different soil types was relatively low on the pixel by pixel basis (R^2^ ranging from 0.266 to 0.441 and RMSD ranging from 0.15 to 0.19), but it was slightly improved when pixel aggregated values were compared.

For the next eight years, use of the SimSphere SVAT model within the “triangle” method was focused mainly on studies demonstrating the use of the “triangle” method in urbanisation applications [65-68]. From a different perspective, [69] used the reviewed here SVAT model and AVHRR data to implement the “triangle” method in the area of Egypt's Nile delta, where they attempted to relate the “triangle” derived maps of M_o_ with the prevalence of diseases such as malaria and filariasis, since the mosquitoes that carry the parasite require standing water for breeding. However, their results unfortunately failed to show a very significant correlation between filariasis infection rate with retrieved M_o_ (R^2^ = 0.37). Subsequent use of the SVAT model in the “triangle” method was carried out by [19], who, for a test region in USA, applied the “triangle” approach using the SVAT model and satellite observations from the high spatial resolution airborne TMS/TIMS (12 m) and the coarse resolution AVHRR (1 km) sensor. A satisfactory agreement to ground-measured energy fluxes was retrieved when using the airborne data, but results from the satellite data were in poor agreement with both the ground data and the airborne data. In the same work, the authors proposed use of the SVAT model in an interpolation scheme for the computation of surface heat fluxes from one time of the day to another, which also allowed for a comparison of results from two satellite views acquired at different times of the day. Last, but not least, as previously stated, a variation of the “triangle” method was recently proposed by [20] for the operational retrieval of surface soil moisture at a 1 km spatial resolution using measurements made by the planned National Polar-orbiting Operational Environmental Satellite System (NPOESS). The approach actually at its present form seem to remote use of the SVAT model, and is instead based on linking microwave-derived soil moisture estimates from the Conical Scanning Microwave Imager/Sounder (CMIS) with parameters derived from optical/infrared (IR) measurements made by the Visible/Infrared Imager Radiometer Sensor Suite (VIIRS). The first step involved the retrieval of soil moisture at 25 km resolution from the CMIS microwave data; and subsequently the microwave-derived soil moisture was related to the higher spatial resolution NDVI, T_s_ and albedo retrievals from VIIRS using the method of [17,31,14]. However, in the [20] is still uncertain how the inversion equations are derived without the use of the SVAT model. [20] demonstrated the potential use of their technique on selected summertime days at a test site located in the USA using data from the Special Sensor Microwave Imager (SSM/I) (25 km spatial resolution) and the AVHRR (1 km spatial resolution). In their analysis, authors discarded all pixels with a high vegetation cover (NDVI > 0.4) since in these areas the uncertainty in the microwave-derived M_o_ estimate is higher due to the perturbing inference of vegetation on the soil-emitted microwave signal. In June 2007, a decision was made for the NPOESS CMIS instrument to be replaced by an alternative, less complex passive microwave instrument, the Microwave Imager Sounder (MIS). VIIRS and MIS are now been planned to be launched on the same NPOESS platforms starting from 2016, and with operations continuing past 2026.

## 4. Discussion and Conclusions

The present article has provided, for the first time, a comprehensive and systematic overview of the use of the 1D SVAT model originally developed by [1], with the most recent implementation of this model being termed “SimSphere”. This overview complements the study of [15] which focused primarily on the description and relative advantages and disadvantages of the “triangle” method by which this SVAT model can be linked to remote sensing data obtained from satellite or airborne instruments.

The present work started with a short description on the SVAT model architecture and of its variations, followed by the overview of the different studies on which it has been employed to date. From the comprehensive overview of the model use presented here it is clear that use of the SVAT model reviewed here has been long-standing and interdisciplinary. The model has been employed in a number of studies either as a stand-alone tool or via its coupling with remote sensing data, and as a suitable tool in assisting the understanding of various phenomena via the study of hypothetical scenarios. The relatively simple model architecture along with the model dependence from a set of easily-obtainable model inputs and its fine time-step of a large array of outputs, consist further assets to the model use, making its initialisation and practical implementation in real testing conditions easier. The review of the model's utilization to date also assisted in the derivation of some critical conclusions with regards to its use.

Exploitation of this SVAT model appears to be currently limited by the inclusion of only one vegetation type per simulation scenario, particularly so with regard to simulating highly fragmented ecosystems. Furthermore, although the SVAT model time-step is satisfactory for simulating the physical processes in the vertical profile of the soil-vegetation-atmosphere continuum, the latter cannot perform simulations for periods longer than one day. This is because SimSphere at its present version does not contain a parameterisation that account for the daily heat loss other than a weak long wave radiation loss, which results in unrealistic conditions if the model is allowed to run over 24-hours.

Until now, verification of the SVAT model performance has been conducted almost solely over agricultural areas (such as corn and/or soybeans), and at test sites located within the continental USA (e.g. [39,43,46]), with only a few analogous studies reported within European or other ecosystems (e.g. [16,25,26]). Additionally, the SVAT model has mainly been implemented on sites of flat terrain and mostly under summer/autumn cloud-free states, which essentially represent near-optimum conditions for its performance. The model validation studies performed so far have also made minimal use of the wide network of eddy covariance flux towers (such as those of FLUXNET; [70]) that have been erected across the continental USA and Europe for many years, and which provide detailed in situ observations of water, momentum and energy fluxes. We must conclude that there is a need to expand the range of model-data intercomparisons to other regions, time periods and comparison datasets.

In terms of sensitivity analyses, only a rather small number of limited studies have been conducted, based on either empirical or local/single factor methods (e.g. [1,16,24]. In these methods, sensitivity of specific model outputs to the model inputs are been typically examined by varying the inputs around their expected ‘nominal’ values and observing the effect on the desired model output - often by calculating the derivative of the output with respect to the input considered. Whilst useful, it is well-known that such approaches have certain limitations, some of the most important being that they do not account for the interactions between model input parameters, allow only small perturbations around each model input parameter base value, and are often rather site-specific. It is our view that further sensitivity analysis studies are required – ideally using global (so-called “variance-based”) sensitivity analysis methods that apportion the output variability to the variability of the input parameters when they vary over their whole uncertainty domain. Reviews of the available GSA methods and of their applications can be found, for example, in [71,72]. GSA methods can deliver global, quantitative sensitivity measures for all model outputs incorporating the influence of all the input parameters over their whole range of variation, including input parameter interactions.

Lastly, implementation of the SVAT model and remotely sensed data within the so-called “triangle” scheme has so far produced results of varying quality with respect to the derived spatially distributed estimates of land surface LE and H fluxes and M_o_. However, particularly when high spatial resolution (i.e. airborne) remote sensing data have been employed, spatially distributed maps of LE and H have been show to be derivable with an accuracy of approximately 25 – 55 Wm^-2^, whilst M_o_ has been estimated to around 0.16 cm cm^-3^ ([14,19,63]). We note that [73], in a comparison study between various remote sensing methodologies for the retrieval of the LE flux, concluded that an upper limit in the estimation accuracy of LE flux (and generally also H flux) were typically of the order of 10 – 20 % (RMSD, varying from 30 to 70 Wm^-2^). However, a more geographically widespread intercomparison between the “triangle” method and in situ measured fluxes is probably now required, particularly so, if a variant of the “triangle” methodology is going to be used operationally by NPOESS and especially if the SVAT model is used in NPOESS methodology, which also remains to be seen.

## Figures and Tables

**Table 1.** Variables and coefficients from the SVAT model of [1] which are shown in figure 2b that is illustrating an Ohm's electrical analogy of the model architecture.

**Variable**	**Name of variable**	**Units**
e_a_	Air vapour pressure in the atmosphere	mbar
e_af_	Leaf-air boundary vapour pressure	mbar
e_L_(T_L_)	The saturation vapour pressure at the temperature of the leaf	mbar
F_w_	Flow of water from soil to leaf	Wm^-2^
H_f_	Foliage sensible heat flux	Wm^-2^
H_g_	Soil sensible heat flux	Wm^-2^
L_e_E_f_	Foliage latent heat flux	Wm^-2^
L_e_E_g_	Soil latent heat flux	Wm^-2^
r_a_	Air resistance in surface layer	sm^-1^
r_af_	Resistance of heat and water vapour flux for interleaf air spaces	sm^-1^
r_ag_	Air resistance between the ground and the interleaf air spaces	sm^-1^
r_b_	Air resistance in transition surface layer	sm^-1^
r_g_	Soil resistance from the substrate	sm^-1^
r_L_	Leaf resistance	sm^-1^
θ_v_	Soil water content of the root zone	cm^3^cm^-3^
θ_vo_	Surface soil water content	cm^3^cm^-3^
ψ_g_	Soil water potential	bar
ψ_L_	Mesophyllic leaf water potential	bar
T_a_	Air temperature of the surface layer	Kelvin
T_af_	Temperature of the interfoliage air spaces	Kelvin
T_g_	Temperature of the ground surface	Kelvin
T_L_	Temperature of the leaf surface	Kelvin
Z_root_	Root resistance	bar(Wm^-2^)^-1^
Z_stem_	Stem resistance	bar(Wm^-2^)^-1^
Z_g_	Soil root surface resistance	bar(Wm^-2^)^-1^
σ_f_	Shielding factor	unitless
